# Plasma indoxyl sulfate levels predict cardiovascular events in patients with mild chronic heart failure

**DOI:** 10.1038/s41598-020-73633-9

**Published:** 2020-10-05

**Authors:** Miki Imazu, Hiroki Fukuda, Hideaki Kanzaki, Makoto Amaki, Takuya Hasegawa, Hiroyuki Takahama, Tatsuro Hitsumoto, Osamu Tsukamoto, Toshisuke Morita, Shin Ito, Masafumi Kitakaze

**Affiliations:** 1 Department of Clinical Research and Development, National Cerebral and Cardiovascular Center, 6-1 Kishibe-Shimmachi, Suita, Osaka 564-8565 Japan; 2 Department of Cardiovascular Medicine, National Cerebral and Cardiovascular Center, 6-1 Kishibe-Shimmachi, Suita, Osaka Japan; 3 Department of Medical Biochemistry, Osaka University Graduate School of Medicine, 2-2 Yamadaoka, Suita, Osaka Japan; 4 Department of Laboratory Medicine, Toho University Omori Medical Center, Tokyo, Japan

**Keywords:** Biomarkers, Cardiology, Medical research

## Abstract

Indoxyl sulfate (IS) is associated with either chronic kidney disease or renal failure, which may predict cardiovascular events via cardiorenal syndrome. The present study aimed to elucidate whether the plasma levels of IS can predict the occurrence of cardiovascular events in patients with chronic heart failure (CHF) and investigate which causes of CHF leading to cardiovascular events are highly influenced by plasma IS levels. We measured the plasma IS levels in 165 patients with CHF [valvular disease: 78, dilated cardiomyopathy: 29, hypertrophic cardiomyopathy (HCM): 25 and others: 33] admitted to our hospital in 2012, and we followed up these patients for more than 5 years (the median follow-up period: 5.3 years). We measured the plasma IS level in 165 patients with CHF, and Kaplan–Meier analyses showed that high plasma IS levels (≥ 0.79 µg/mL, the median value) could predict the occurrence of cardiovascular events, i.e., cardiovascular death or rehospitalization due to the worsening of CHF. The sub-analyses showed that the high IS level could predict cardiovascular events in patients with CHF due to HCM and that the plasma IS levels were closely associated with left ventricular (LV) dimension, LV systolic dysfunction, and plasma B-type natriuretic peptide levels, rather than LV diastolic dysfunction. Plasma IS level predicts cardiovascular events in patients with CHF, especially those with HCM along with cardiac dysfunction. Besides, IS may become a proper biomarker to predict cardiovascular events in patients with CHF.

## Introduction

In patients with chronic heart failure (CHF), dysfunctions of other organs, including kidney, liver, and intestine, rather than heart were observed^1^, which might increase uremic toxins. Uremic toxins play a substantial role in cardiovascular injury^2^, and indoxyl sulfate (IS) is surmised to be the most abundant and potent uremic toxin^3^. Several lines of evidence have shown that renal dysfunction increases IS levels^4^, leading not only the renal function getting affected^5,6^ but also cardiac function^7^. We have previously reported the increase of plasma IS levels in CHF patients with preserved renal function as well as the removal of IS using AST-120, an adsorbent of uremic toxins in the gut, restoring the left ventricular (LV) systolic and diastolic function^8^. This clinical observation has been confirmed in a pacing-induced HF canine model; the plasma IS levels increase along with the progression of HF, and AST-120 improves LV dysfunction^9^. Indeed, IS increases the expression and activation of extracellular signal-regulated kinase (ERK), P38MAP kinase, and nuclear factor kappa light chain enhancer of activated B cells (NF-kB), which may affect cardiac remodeling^10,11^. Furthermore, IS activates renin receptors^12^, thereby activating angiotensin receptors^13^. Either of these deleterious sequelae may affect cardiomyocytes, cardiac fibroblasts, and cardiac endothelial cells^14^ and lead to cardiovascular dysfunction. These results support the theory that plasma IS may become a novel biomarker for the prediction of cardiovascular events in patients with CHF. Hence, it is essential to investigate which causes of CHF leading to cardiovascular events are highly influenced by the plasma IS levels.

To test this theory, we retrospectively and consecutively enrolled patients with CHF who were hospitalized for the worsening of CHF, initial onset of acute HF (AHF), or precise examination in our hospital at 2012, and we followed up with these patients for more than 5 years.

## Results

**Figure 1:**
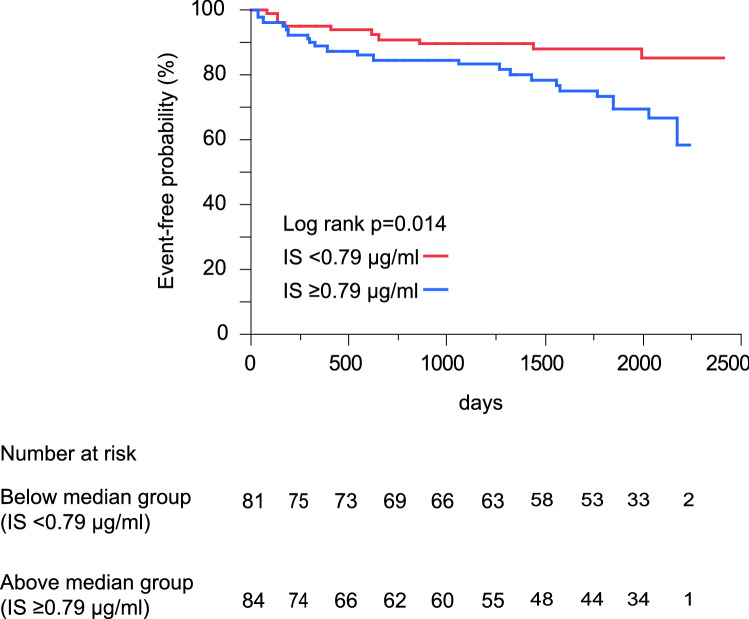
High plasma IS levels predict cardiovascular events in patients with CHF—the event-free survival curves based on Kaplan–Meier estimator in CHF patients with high and low plasma IS levels. The cutoff value for high and low IS levels is the median value of CHF patients. The cardiovascular events were predicted in CHF patients with high plasma IS levels compared with those with low IS levels.

The characteristics of the 165 included patients with CHF are shown in Table 1. The plasma IS levels were high in patients with CHF compared with control subjects in our previous study^8^. The median value of the plasma IS levels was 0.79 µg/mL, and we divided the present patients according the IS value of ≥ 0.79 (high IS level) and < 0.79 (low IS level) µg/mL. Figure 1 shows the Kaplan–Meyer survival curves for patients with CHF with both high and low plasma IS levels, and the rate of cardiovascular events in patients with high plasma IS levels was higher than that in patients with low plasma IS levels. Cardiovascular events occurred in higher percentage of patients with high plasma IS levels (cardiovascular death: 4 of 84 [4.8%] and the rehospitalization due to worsening of HF: 19 of 84 [22.6%]) than with low plasma IS levels (1 of 81 [1.2%] and 9 of 81 [11.1%]). The importance of the plasma IS levels on cardiovascular events is confirmed after the adjustment of other risk factors (Table 2).

**Table 1:** Patients’ characteristics.

	Total	Valvular disease	DCM	HCM	Others	P value
n	165	78	29	25	33	
**Demographic data**						
Age, year	65 (51–73)	69 (63–76)	55 (38–65)	65 (52–69)	56 (40–73)	< 0.001
Women/men	81/84	46/32	7/22	16/9	12/21	0.002
New York Heart Association class III–IV (%)	9 (5)	3 (4)	1 (3)	3 (12)	2 (6)	0.572
**History**						
Hypertension (%)	79 (48)	43 (55)	10 (34)	5 (20)	21 (64)	0.002
Diabetes mellitus (%)	44 (27)	18 (24)	12 (41)	4 (16)	10 (30)	0.159
Stroke (%)	19 (12)	9 (12)	3 (10)	2 (8)	5 (15)	0.857
Atrial fibrillation (%)	52 (32)	23 (29)	9 (31)	8 (32)	12 (36)	0.916
**Physical findings**						
Systolic blood pressure (mmHg)	111 ± 15	116 ± 14	105 ± 16	106 ± 15	109 ± 16	0.002
Heart rate (beats/min)	69 ± 12	66 ± 12	73 ± 11	67 ± 10	72 ± 14	0.039
Body mass index (kg/m^2^)	23 (20–25)	22 (20–24)	23 (21–27)	22 (21–25)	22 (20–26)	0.198
**Medications**						
β-Blockers (%)	97 (59)	25 (32)	26 (90)	20 (80)	26 (79)	< 0.001
Angiotensin-converting enzyme inhibitors or angiotensin II receptor blockers (%)	95 (57)	41 (53)	23 (79)	8 (32)	23 (70)	0.008
Loop diuretics (%)	75 (45)	31 (40)	20 (69)	6 (24)	18 (55)	0.004
Aldosterone antagonists (%)	57 (35)	15 (19)	20 (69)	6 (24)	16 (48)	< 0.001
Statins (%)	57 (35)	30 (38)	7 (24)	8 (32)	12 (36)	0.565
**Laboratory data**						
IS (μg/ml)	0.79 (0.44–1.24)	0.77 (0.49–1.13)	0.77 (0.43–1.20)	0.94 (0.29–1.54)	0.87 (0.44–1.26)	0.896
Albumin (g/dl)	4.3 ± 0.4	4.3 ± 0.3	4.3 ± 0.4	4.3 ± 0.4	4.1 ± 0.4	0.168
Hemoglobin (g/dl)	13.1 ± 1.7	12.6 ± 1.5	14.3 ± 1.4	13.2 ± 1.7	13.3 ± 2.1	< 0.001
BUN (mg/dl)	17 (14–21)	17 (15–21)	15 (14–20)	19 (15–25)	17 (14–21)	0.090
Creatinine (mg/dl)	0.8 (0.7–0.9)	0.7 (0.6–0.9)	0.9 (0.8–1.0)	0.7 (0.6–0.9)	0.8 (0.7–1.0)	0.002
eGFR (ml/min/1.73 m^2^)	69 (58–79)	68 (59–79)	69 (57–78)	69 (59–75)	71 (54–82)	0.977
Uric acid (mg/dl)	5.7 (4.7–7.3)	5.5 (4.6–6.8)	6.9 (5.4–8.8)	5.4 (4.9–6.5)	6.4 (5.2–8.0)	0.022
BNP (pg/ml)	106 (54–249)	91 (52–177)	155 (54–324)	291 (90–454)	106 (49–240)	0.007
**Echocardiography data**						
n	165	78	29	25	33	
LVEDD (mm)	55 ± 12	51 ± 9	67 ± 7	47 ± 11	59 ± 12	< 0.001
LVESD (mm)	38 (29–53)	33 (27–38)	60 (54–64)	26 (23–42)	49 (37–57)	< 0.001
%FS (%)	30 (16–39)	36 (30–40)	9 (8–15)	38 (21–44)	18 (14–24)	< 0.001
LVEF (%)	53 (30–63)	63 (53–67)	23 (18–31)	63 (43–68)	32 (23–43)	< 0.001
E/A	1.0 (0.7–1.8)	1.0 (0.6–1.9)	1.5 (0.7–2.5)	1.0 (0.8–1.6)	0.8 (0.7–1.6)	0.490
DcT (ms)	203 (164–254)	223 (134–280)	162 (123–209)	214 (173–312)	187 (146–248)	< 0.001
E/e′	12 (9–17)	14 (10–18)	11 (9–15)	15 (11–18)	10 (8–14)	0.015
IVC, mm	13 (10–16)	13 (11–16)	14 (10–17)	12 (9–15)	12 (8–19)	0.553
**Central hemodynamics data**						
n	165	78	29	25	33	
Mean RA pressure (mmHg)	4 (2–5)	4 (2–6)	3 (2–4)	4 (3–5)	3 (2–5)	0.198
Mean PA pressure (mmHg)	18 (14–23)	18 (14–22)	16 (13–24)	19 (15–25)	17 (12–23)	0.505
Mean PCW pressure (mmHg)	11 (7–14)	11 (8–13)	8 (5–15)	12 (9–16)	10 (5–14)	0.114
LV end-diastolic pressure (mmHg)	14 (8–20)	12 (10–18)	15 (7–22)	21 (15–24)	10 (7–15)	< 0.001
Cardiovascular events (%)	33 (20)	9 (12)	6 (21)	8 (32)	10 (30)	0.047

Date are expressed as numbers of patients (n), percentages and median values (25th–75th percentiles) or mean ± SD.

*DCM* dilated cardiomyopathy, *HCM* hypertrophic cardiomyopathy, *IS* indoxyl sulfate, *BUN* blood urea nitrogen, *eGFR* estimated glomerular filtration rate, *BNP* B-type natriuretic peptide, *LVEDD* left ventricular (LV) end-diastolic dimension; *LVESD* LV end-systolic dimension, *FS* fractional shortening, *LVEF* LV ejection fraction, *E* Peak velocity of early diastolic filling, *A* Late diastolic filling due to atrial contraction, *DcT* deceleration time, *e'* early diastolic mitral annular tissue velocity, *IVC* inferior vena cava, *RA* right atrial, *PA* pulmonary artery, *PCW* pulmonary capillary wedge.

**Table 2:** Predictive values of plasma IS levels on cardiovascular events during 5 years.

	Hazard ratios (95% confidence interval)	P value
Unadjusted	1.84 (1.282–2.513)	0.002
Adjusted for eGFR	1.72 (1.116–2.414)	0.009
Adjusted for eGFR, age, sex	1.81 (1.206–2.575)	0.006
Adjusted for age, sex, and BUN	1.84 (1.247–2.576)	0.003
Adjusted for age, sex, and BNP	1.92 (1.310–2.683)	0.002

Abbreviations are same as in Table 1.

To investigate which causes of CHF leading to cardiovascular events are highly influenced by the plasma IS levels, we performed a sub-analysis in each group categorized by the cause of CHF, i.e., valvular disease, dilated cardiomyopathy (DCM), hypertrophic cardiomyopathy (HCM), and the others. We found that plasma IS levels especially affected the cardiovascular parameters and cardiovascular events in the patients with CHF with HCM (Supplementary Tables S1–S3), although the number of each category is too small for precise analysis.

## Discussion

The major findings of this study were as follows: (1) the plasma IS levels in the patients with CHF predicted the cardiovascular events of either cardiovascular death or rehospitalization due to the worsening of HF; (2) the plasma IS level appropriately predicted the cardiovascular events in patients with CHF accompanied by HCM, whereas the other causes in the sub-analyses for the causes of CHF were not involved; and (3) the plasma IS level was closely associated with the LV systolic function in CHF patients with HCM.

### Influences of the plasma IS levels on prediction of cardiovascular events in patients with CHF

First, it was essential to indicate whether CHF per se increases plasma IS level independent of eGFR or secondary renal dysfunction attributable to the pathophysiology of CHF (e.g., low perfusion to the kidney or renal edema) may increase the plasma IS level. Our previous study revealed that the plasma IS levels increased in patients with CHF compared with control subjects with a comparable eGFR^8^, suggesting that CHF per se increases the plasma IS levels. As IS is released to urinary tubules via organic anion transporter 1, 3 (OAT1 and OAT3) and is excreted to urine via OAT4^15^, the pathophysiology of CHF increases plasma IS level, which may be attributable to direct effect of OATs without influencing eGFR. Indeed, in the present study, eGFR did not decrease in CHF patients (Table 1). CHF may activate the cytokine and renin–angiotensin systems, and either cytokine or angiotensin II is reported to inactivate OAT^16–18^. Neurohumoral modulation in the pathophysiology of CHF may directly increase the plasma IS level independent of secondary renal dysfunction. Furthermore, malfunction of intestine or liver in patients with CHF may increase the plasma IS level.

The next issue is whether IS directly affects the tissues or cells in cardiovascular systems. Intriguingly, IS can activate the renin receptors^12^ as well as cause angiotensin receptor activation^13^, which may contribute to cardiac hypertrophy and fibrosis. Furthermore, IS increases the expression of NF-kB^11^, enhancing the inflammations, and IS decreases the NOS activity^19^, which may largely contribute to the formation of CHF in the experimental and clinical studies. Ultimately, IS increases oxidative stress^20^, worsening the pathophysiology of HF, it decreases erythropoietin (EPO) level^21^ despite the cardioprotective effects of EPO^22^, and it decreases the expression of Klotho in kidne^23,24^. Decreasing the expression of Klotho may increase fibroblast growth factor 23 (FGF23), and the increased FGF23 level may cause cardiac hypertrophy and CHF. One or some of these factors may explain the cardiac deleterious effects of IS observed in the present study.

IS may be closely associated with the pathophysiology of CHF, and IS may worsen the severity of CHF. However, in the present study, it remained unknown whether IS is deleterious or beneficial for the severity of CHF because CHF increases the plasma BNP level, and increased BNP is beneficial for CHF due to the cardioprotective effects of BNP. However, several lines of evidence, including our earlier studies, showed that the removal of IS using AST-120 exerts beneficial effects on the pathophysiology of CHF in the clinical^8,25^ and experimental^25–27^ studies, suggesting that the elevation of the plasma IS level in patients with CHF may contribute to the worsening of CHF.

### The role of plasma IS levels in the various causes of CHF

In the present study, we showed that IS plays a pivotal role in the progression of patients with CHF, especially those with HCM; besides, LV systolic dysfunction in CHF patients with HCM was found to associate with plasma IS levels. We found that RA pressure had a good correlation with plasma IS level in HCM patients although the sample size is too small to draw the definite conclusion seen in the supplemental data. The patients with HCM suffers from LV diastolic dysfunction, causing the elevation of RA pressure. The increase in RA pressure provides renal congestion and dysfunction followed by the elevation of the plasma IS levels. This is one of the scenarios that RA pressure had a correlation with plasma IS level in HCM patients in our data. However, a previous study showed that blood IS level correlated well with the severity of LV diastolic dysfunction in patients with DCM^28^. These results seem to be inconsistent with ours; however, the discrepancy may be attributable to the patients’ characteristics. Although either DCM or HCM does not necessarily provoke symptomatic CHF, the patients with CHF with DCM or HCM in the present study experienced hospitalization due to AHF, and cardiovascular events included recurrent worsening HF or cardiovascular death, which revealed that severity of CHF in DCM or HCM patients was more than that found a previous study^28^. The role of plasma IS as a biomarker for the severity of CHF in patients with severe DCM at the end-stage of CHF may be blunted by the other deleterious neurohumoral factors, such as tumor necrosis factor alpha (TNF-α). This may demonstrate that the sensitivity to detect the severity of HF by the plasma IS levels may be limited in patients with severe CHF.

### Limitations of the present study

Firstly, daily dietary situation may affect the plasma IS level, because intake of the food containing tryptophan might affect the IS level. However, as we measured the blood samples after a 12-h fast, dietary situation may not have influenced the IS level in the present study.

Secondly, this study was performed as a single-center study, and the differences among the hospitals should have been taken into account. However, since the diagnosis and treatment of CHF are undertaken according to the guidelines of the treatment of HF in Japan and are very similar to American College of Cardiology/American Heart Association (ACC/AHA) or European Society of Cardiology guidelines, the patients’ characteristics were found to be identical worldwide.

Thirdly, the number of patients in each category of causes of CHC, including HCM and DCM, was not remarkable; therefore, further studies should be conducted to verify our findings.

Lastly, although our hospital is one of high-volume centers for the HF patients, we measured the plasma IS level in the limited number of the patients because long-term follow-up is difficult. However, because of the positive results of that the plasma IS levels increase and predict clinical outcomes in patients with HF, plasma IS levels may become a biomarker of HF such as plasma BNP levels.

### The future direction from the present study

The present study strongly suggests that the measurement of the plasma IS level in advance predicts following cardiovascular events in CHF patients, especially those CHF patients with HCM. Furthermore, we need to test the effectiveness of AST-120 for the improvement of pathophysiology of CHF.

## Methods

### Study subjects

We included 165 patients who were admitted to our hospital for the treatment of worsened CHF or the initial onset of AHF from January to December 2012. The causes of CHF were categorized as valvular disease (n = 78), DCM (n = 29), HCM (n = 25), and others (n = 33) such as hypertensive heart disease (n = 12), ischemic cardiomyopathy (n = 4), amyloidosis (n = 5) and sarcoidosis (n = 2). Patients were included if they were stable for CHF and had eGFR more than 40 ml/min/1.73 m^2^; the patients showed a New York Heart Association functional class of either I, II or III at the discharge. Diagnosis of CHF was conducted based on the Framingham criteria^15^, and the patients were excluded if they had CHF with more than the middle-stage chronic kidney disease (CKD) (Stage 3 CKD)^16^. We sampled the blood in a stable chronic phase of CHF during hospitalization. After the discharge following the appropriate treatment of HF, we followed up with these patients for more than 5 years and searched for the cardiovascular events of either cardiovascular death or the rehospitalization due to worsening of HF.

### Measurement of biomarkers

Blood for the serum was collected in tubes; the serum was then separated, and blood for the plasma was collected in ethylenediaminetetraacetic acid (EDTA) tubes; the plasma was then separated and then samples for IS were frozen in plastic tubes at − 80 °C until analysis. We measured IS levels by internal-surface reversed-phase high-performance liquid chromatography (HPLC), with a HPLC system (Shimadzu, Kyoto, Japan), as previously described^29^. The Japanese-specific eGFR was calculated as follows: [194 × Serum creatinine^−1.094^ × Age^−0.287^ × (0.739 for females)]^18^.

### Echocardiography

We measured and calculated LV systolic and diastolic dimensions according to the Guidelines of American Society of Echocardiography^19^. A comprehensive echo-Doppler assessment was performed according to the Guidelines of American Society of Echocardiography. Whereas LV ejection fraction (EF) was obtained using the Simpson biplane method or Teichholz's formula, fractional shortening (FS) was evaluated using the following formula: FS = 100 × (LVEDD − LVESD)/LVEDD, where LVEDD is the LV end-diastolic dimension and LVESD is the LV end-systolic dimension. LV E/e′ was also measured as the ratio of peak velocity of early transmitral diastolic filling by echocardiography to early diastolic mitral annular velocity by tissue Doppler echocardiography as LV diastolic function.

### Cardiac catheterization for hemodynamic data

We performed right and left heart catheterizations on the study participants and prospectively collected clinical data as described before^30^. We performed a standard right heart catheterization via either the internal jugular vein or the femoral vein by using a Swan-Ganz catheter (Goodman, Tokyo, Japan). A standard left heart catheterization was performed via either the radial artery or femoral artery by using an angled pigtail catheter (Terumo, Tokyo, Japan).

Using the right and left heart catheterization data, we obtained the cardiac hemodynamic parameters, including the right atrial pressure, pulmonary artery pressure, pulmonary capillary wedge pressure and LV end-diastolic pressure.

### Statistical analysis

Continuous data were presented as median with interquartile range (IQR), where IQRs were presented in terms of 25th and 75th percentiles or mean ± standard deviation. Categorical data were presented as frequencies (%). Statistical significance between the groups was evaluated by One-way ANOVA or Kruskal–Wallis test. The event-free survival curves were estimated using the Kaplan–Meier method. Between-group differences in survival were assessed using the log-rank test. HRs with 95% confidence interval were calculated using Cox proportional hazards regression, including those for the subgroup analyses. The proportional hazards assumption was graphically investigated based on the Schoenfeld residuals over time. Univariate linear regression analysis was performed to assess the relationships between IS and the other variables. In the present study, all tests were two-tailed; P < 0.05 was considered statistically significant. The analyses were performed with the JMP 8.0.2 software for Windows (SAS Institute Inc., Cary, NC, USA).

### Ethics approval

This study was approved by the Institutional Ethics Committee of the National Cerebral and Cardiovascular Center (R19098; Osaka, Japan) and was conducted in accordance with the principles enshrined in the 2013 Declaration of Helsinki and Japanese ethical guidelines for clinical research. All the patients a signed written informed consent form.

## Supplementary information

Supplementary Information.

## Data Availability

We declare that all the data supporting the findings of this study are available within the paper.

## Abbreviations

CHF: Chronic heart failure
IS: Indoxyl sulfate
LV: Left ventricular
AHF: Acute heart failure
DCM: Dilated cardiomyopathy
HCM: Hypertrophic cardiomyopathy
eGFR: Estimated glomerular filtration rate
CKD: Chronic kidney disease
EF: Ejection fraction
FS: Fractional shortening
LVEDD: LV end-diastolic dimension
LVESD: LV end-systolic dimension
BNP: B-type natriuretic peptide
OAT: Organic anion transporter

## References

[1] Silverberg DS, Wexler D, Blum M, Iaina A (2003). The cardio renal anemia syndrome: Correcting anemia in patients with resistant congestive heart failure can improve both cardiac and renal function and reduce hospitalizations. Clin. Nephrol..

[2] Lekawanvijit S, Kompa AR, Wang BH, Kelly DJ, Krum H (2012). Cardiorenal syndrome: The emerging role of protein-bound uremic toxins. Circ. Res..

[3] Duranton F (2012). Normal and pathologic concentrations of uremic toxins. J. Am. Soc. Nephrol. JASN.

[4] Lin C-J, Wu V, Wu P-C, Wu C-J (2015). Meta-analysis of the associations of p-cresyl sulfate (PCS) and indoxyl sulfate (IS) with cardiovascular events and all-cause mortality in patients with chronic renal failure. PLoS ONE.

[5] Niwa T (1997). Indoxyl sulfate and progression of renal failure: Effects of a low-protein diet and oral sorbent on indoxyl sulfate production in uremic rats and undialyzed uremic patients. Miner. Electrolyte Metab..

[6] Niwa T, Ise M (1994). Indoxyl sulfate, a circulating uremic toxin, stimulates the progression of glomerular sclerosis. J. Lab. Clin. Med..

[7] Barreto FC (2009). Serum indoxyl sulfate is associated with vascular disease and mortality in chronic kidney disease patients. Clin. J. Am. Soc. Nephrol..

[8] Imazu M (2017). A pathophysiological role of plasma indoxyl sulfate in patients with heart failure. Int. J. Gerontol..

[9] Asanuma H (2019). AST-120, an adsorbent of uremic toxins, improves the pathophysiology of heart failure in conscious dogs. Cardiovasc. Drugs Ther..

[10] Lekawanvijit S (2010). Does indoxyl sulfate, a uraemic toxin, have direct effects on cardiac fibroblasts and myocytes?. Eur. Heart J..

[11] Shimizu H, Yisireyili M, Higashiyama Y, Nishijima F, Niwa T (2013). Indoxyl sulfate upregulates renal expression of ICAM-1 via production of ROS and activation of NF-kappaB and p53 in proximal tubular cells. Life Sci..

[12] Yisireyili M (2014). Indoxyl sulfate-induced activation of (pro)renin receptor promotes cell proliferation and tissue factor expression in vascular smooth muscle cells. PLoS ONE.

[13] Shimizu H, Saito S, Higashiyama Y, Nishijima F, Niwa T (2013). CREB, NF-kappaB, and NADPH oxidase coordinately upregulate indoxyl sulfate-induced angiotensinogen expression in proximal tubular cells. Am. J. Physiol. Cell Physiol..

[14] Rana I (2015). Contribution of microRNA to pathological fibrosis in cardio-renal syndrome: Impact of uremic toxins. Physiol. Rep..

[15] Enomoto A, Niwa T (2007). Roles of organic anion transporters in the progression of chronic renal failure. Ther. Apheresis Dial..

[16] Le Vee M, Gripon P, Stieger B, Fardel O (2008). Down-regulation of organic anion transporter expression in human hepatocytes exposed to the proinflammatory cytokine interleukin 1β. Drug Metab. Dispos..

[17] Favretto G (2017). Role of organic anion transporters in the uptake of protein-bound uremic toxins by human endothelial cells and monocyte chemoattractant protein-1 expression. J. Vasc. Res..

[18] Duan P, Li S, You G (2010). Angiotensin II inhibits activity of human organic anion transporter 3 through activation of protein kinase Cα: Accelerating endocytosis of the transporter. Eur. J. Pharmacol..

[19] Yu M, Kim YJ, Kang DH (2011). Indoxyl sulfate-induced endothelial dysfunction in patients with chronic kidney disease via an induction of oxidative stress. Clin. J. Am. Soc. Nephrol. CJASN.

[20] Dou L (2007). The uremic solute indoxyl sulfate induces oxidative stress in endothelial cells. J. Thromb. Haemost. JTH.

[21] Chiang CK, Tanaka T, Inagi R, Fujita T, Nangaku M (2011). Indoxyl sulfate, a representative uremic toxin, suppresses erythropoietin production in a HIF-dependent manner. Lab. Investig. J. Tech. Methods Pathol..

[22] Hirata A (2006). Erythropoietin enhances neovascularization of ischemic myocardium and improves left ventricular dysfunction after myocardial infarction in dogs. J. Am. Coll. Cardiol..

[23] Adijiang A, Niwa T (2010). An oral sorbent, AST-120, increases Klotho expression and inhibits cell senescence in the kidney of uremic rats. Am. J. Nephrol..

[24] Sun CY, Chang SC, Wu MS (2012). Suppression of Klotho expression by protein-bound uremic toxins is associated with increased DNA methyltransferase expression and DNA hypermethylation. Kidney Int..

[25] Niwa T (2011). Role of indoxyl sulfate in the progression of chronic kidney disease and cardiovascular disease: Experimental and clinical effects of oral sorbent AST-120. Ther. Apheresis Dial..

[26] Asanuma H (2019). AST-120, an adsorbent of uremic toxins, improves the pathophysiology of heart failure in conscious dogs. Cardiovasc. Drugs Ther..

[27] Nishikawa M (2015). AST-120 ameliorates lowered exercise capacity and mitochondrial biogenesis in the skeletal muscle from mice with chronic kidney disease via reducing oxidative stress. Nephrol. Dial. Transplant..

[28] Shimazu S (2013). Association between indoxyl sulfate and cardiac dysfunction and prognosis in patients with dilated cardiomyopathy. Circ. J..

[29] Niwa T, Takeda N, Tatematsu A, Maeda K (1988). Accumulation of indoxyl sulfate, an inhibitor of drug-binding, in uremic serum as demonstrated by internal-surface reversed-phase liquid chromatography. Clin. Chem..

[30] Imazu M (2017). Use of serum fibroblast growth factor 23 vs. plasma B-type natriuretic peptide levels in assessing the pathophysiology of patients with heart failure. Hypertens. Res..

