# Supplementary material for: Plasma indoxyl sulfate levels predict cardiovascular events in patients with mild chronic heart failure
Source: Sci Rep. 2020 Oct 5;10:16528. doi: 10.1038/s41598-020-73633-9 (PMC7536212; doi:10.1038/s41598-020-73633-9)
Supplement: Supplementary file 1 — Supplementary Information. [file 41598_2020_73633_MOESM1_ESM.docx]

**Plasma Indoxyl Sulfate Levels Predict Cardiovascular Events**

**in Patients with Mild Chronic Heart Failure**

Miki Imazu^1^, Hiroki Fukuda^1^, Hideaki Kanzaki^2^, Makoto Amaki^2^, Takuya Hasegawa^2^, Hiroyuki Takahama^2^, Tatsuro Hitsumoto^1^, Osamu Tsukamoto^3^, Toshisuke Morita^4^, Shin Ito^1^, Masafumi Kitakaze*^1^

^1^Department of Clinical Research and Development and ^2^Department of Cardiovascular Medicine, National Cerebral and Cardiovascular Center, 6-1 Kishibe-Shimmachi, Suita Osaka, Japan, ^3^Department of Medical Biochemistry, Osaka University Graduate School of Medicine, 2-2 Yamadaoka Suita, Osaka, Japan, ^4^Department of Laboratory Medicine, Toho University Omori Medical Center, Tokyo, Japan.

*Corresponding author, E-mail Address: [kitakaze@zf6.so-net.ne.jp](mailto:kitakaze@zf6.so-net.ne.jp)

**Supplementary Table S1. Predictive values of plasma IS levels on cardiovascular events during 5 years**

|  |  |  | Hazard Ratios (95% Confidence Interval) | P value |
| --- | --- | --- | --- | --- |
| DCM | |  |  |  |
|  | Unadjusted |  | 0.87 (0.165-2.529) | 0.831 |
|  | Adjusted for eGFR |  | 0.79 (0.146-2.429) | 0.730 |
|  | Adjusted for eGFR, age, sex |  | 0.79 (0.171-2.395) | 0.703 |
| HCM | |  |  |  |
|  | Unadjusted |  | 2.61 (1.303-5.426) | 0.008 |
|  | Adjusted for eGFR |  | 2.59 (1.111-6.277) | 0.028 |
|  | Adjusted for eGFR, age, sex |  | 2.91 (1.202-7.498) | 0.019 |
| Valvular disease | |  |  |  |
|  | Unadjusted |  | 1.98 (0.997-3.340) | 0.051 |
|  | Adjusted for eGFR |  | 1.84 (0.858-3.232) | 0.105 |
|  | Adjusted for eGFR, age, sex |  | 1.98 (0.913-3.579) | 0.078 |
| Others | |  |  |  |
|  | Unadjusted |  | 2.11 (0.729-5.886) | 0.163 |
|  | Adjusted for eGFR |  | 1.95 (0.663-5.519) | 0.215 |
|  | Adjusted for eGFR, age, sex |  | 1.80 (0.526-5.716) | 0.330 |

Abbreviations are same as in Table 1.

**Supplementary Table S2. Univariate and multivariable analyses of plasma IS levels in the CHF patients with HCM**

|  | Univariate analyses of plasma IS levels in the CHF patients with HCM | | | |
| --- | --- | --- | --- | --- |
|  |  | Regression coefficient, pg/ml | 95%CI | P value |
| Echocardiography data | |  |  |  |
|  | LVEDD | 0.050 | 0.017, 0.082 | 0.004 |
|  | LVESD | 0.036 | 0.012, 0.060 | 0.005 |
|  | %FS | -0.026 | -0.050, -0.001 | 0.044 |
|  | LVEF | -0.037 | -0.055, -0.019 | 0.0003 |
|  | E/A | 0.014 | -0.549, 0.578 | 0.958 |
|  | DcT | -0.002 | -0.005, 0.002 | 0.307 |
|  | E/e' | 0.066 | -0.036, 0.167 | 0.192 |
|  | IVC | 0.102 | 0.012, 0.192 | 0.029 |
| Central Hemodynamics data | | |  |  |
|  | Mean RA pressure | 0.204 | 0.068, 0.339 | 0.005 |
|  | Mean PA pressure | 0.042 | -0.021, 0.105 | 0.181 |
|  | Mean PCW pressure | 0.058 | -0.025, 0.142 | 0.161 |
|  | LV end-diastolic pressure | -0.002 | -0.060, 0.057 | 0.955 |
| Laboratory data | |  |  |  |
|  | eGFR | -0.033 | -0.055, -0.011 | 0.006 |
|  | BNP | 0.000 | -0.001, 0.001 | 0.885 |
|  | Multivariable analyses of plasma IS levels in the CHF patients with HCM | | | |
|  |  | Regression coefficient, pg/ml | 95%CI | P value |
|  | LVEDD | 0.091 | -0.053, 0.235 | 0.198 |
|  | LVESD | -0.093 | -0.213, 0.027 | 0.117 |
|  | LVEF | -0.066 | -0.120, -0.012 | 0.021 |
|  | IVC | 0.016 | -0.083, 0.116 | 0.733 |
|  | Mean PA pressure | -0.012 | -0.072, 0.049 | 0.687 |
|  | eGFR | -0.024 | -0.044, -0.003 | 0.029 |

Abbreviations are same as in Table 1. CHF, chronic heart failure.

**Supplementary Table S3.**

**Hazard ratios with 95% confidence intervals for cardiovascular events during 5 years**

|  |  |  | Hazard Ratios (95% Confidence Interval) | P value |
| --- | --- | --- | --- | --- |
| HCM | |  |  |  |
|  | IS |  | 2.52 (1.10-6.04) | 0.029 |
|  | eGFR |  | 1.00 (0.92-1.06) | 0.899 |
|  | BNP |  | 1.00 (0.99-1.00) | 0.377 |
|  | age |  | 1.02 (0.96-1.10) | 0.489 |

Abbreviations are same as in Table 1.
